# A fusion approach to improve accuracy and estimate uncertainty in cuffless blood pressure monitoring

**DOI:** 10.1038/s41598-022-12087-7

**Published:** 2022-05-13

**Authors:** Cederick Landry, Sean D. Peterson, Arash Arami

**Affiliations:** 1 Mechanical and Mechatronics Engineering Department, University of Waterloo, Waterloo, ON Canada; 2 Toronto Rehabilitation Institute (KITE), University Health Network, Toronto, ON Canada

**Keywords:** Biomedical engineering, Hypertension

## Abstract

A substantial barrier to the clinical adoption of cuffless blood pressure (BP) monitoring techniques is the lack of unified error standards and methods of estimating measurement uncertainty. This study proposes a fusion approach to improve accuracy and estimate prediction interval (PI) as a proxy for uncertainty for cuffless blood BP monitoring. BP was estimated during activities of daily living using three model architectures: nonlinear autoregressive models with exogenous inputs, feedforward neural network models, and pulse arrival time models. Multiple one-class support vector machine (OCSVM) models were trained to cluster data in terms of the percentage of outliers. New BP estimates were then assigned to a cluster using the OCSVMs hyperplanes, and the PIs were estimated using the BP error standard deviation associated with different clusters. The OCSVM was used to estimate the PI for the three BP models. The three BP estimations from the models were fused using the covariance intersection fusion algorithm, which improved BP and PI estimates in comparison with individual model precision by up to 24%. The employed model fusion shows promise in estimating BP and PI for potential clinical uses. The PI indicates that about 71%, 64%, and 29% of the data collected from sitting, standing, and walking can result in high-quality BP estimates. Our PI estimator offers an effective uncertainty metric to quantify the quality of BP estimates and can minimize the risk of false diagnosis.

## Introduction

Non-invasive, cuff-based, 24-h ambulatory blood pressure (BP) monitoring is recommended for accurate diagnosis of hypertension^1^, a condition that affects 1.13 billion people worldwide^2^. Though cuff-based ambulatory BP measurement has been shown to result in better treatment management^3^, patient compliance is relatively low due to the discomfort and disruption of sleep and activities of daily living caused by the intermittently inflating cuffs^4^.

Cuffless BP estimation, a method that relies on the information encoded in proxy physiological signals, generally electrocardiography (ECG) and/or photoplethysmography (PPG), coupled with a surrogate model, has the potential to continuously monitor BP less invasively than traditional cuff-based systems. There are two main cuffless BP estimation methods to estimate systolic BP (SBP) and diastolic BP (DBP)^5^: the methods based on (i) pulse transit time (PTT) or pulse arrival time (PAT), and (ii) feature extraction from the PPG and/or ECG waveforms. Recently, an alternative method has been developed that uses a nonlinear autoregressive model with exogenous inputs (NARX) to estimate the complete BP waveform that has, to date, been demonstrated on bedridden patients^6^, and during activities of daily living^7^. Despite the advantages of cuffless BP monitoring, it has yet to be adopted clinically because of concerns over accuracy of the methods, minimal validation on clinically-relevant data sets, and the lack of a quantifiable uncertainty metric.

In an effort to overcome the first barrier, BP interventions have been implemented to increase the range of BPs upon which estimation models are trained. Many different interventions have been used, such as mental arithmetic^8–10^, cold pressor test^9–11^, nitroglycerin^9^, low-intensity exercise^12,13^, static handgrip exercise^7,14,15^, Valsalva maneuver^7,14,15^ and slow breathing^9,10^. However, it is difficult to know how the BP changes imposed by the interventions reflect natural changes in BP during daily life.

In another effort to overcome the accuracy barrier, sensor fusion has been employed to improve BP estimates. For example, using multiple bioimpedance sensors, Heydari et al. were able to improve PAT-based BP estimates by 3% over PPG-based PAT^16^. Miao et al. demonstrated that the extracted features from ECG and two pulse pressure waveform (or two PPG) signals using their algorithm for BP estimation lead to 1.5% improvement in accuracy when compared with other state of the art algorithms^17,18^. Other fusion approaches have been introduced, such as using a gyroscope or machine learning approaches to assess signal quality to assess signal quality during at-home monitoring^19^, but those have yet to be validated. Track fusion, which combines multiple BP estimates to improve accuracy was shown by Liu et al. to improve model precision by at least 2.5% when fusing 21 models^20^.

While sensor and track fusion have been employed to improve estimate accuracy in cuffless BP monitoring, to date there has been no consideration of the third barrier to clinical adoption, namely uncertainty quantification. The uncertainty in cuff-based measurements has long been of concern for practitioners^21^, leading to the development of methods for estimating it^4^. The standard for expressing uncertainty for measurement devices^22^ states that it should apply to a broad span of conditions. In practice, however, the uncertainty is often obtained from the measurement error with a series of measurements under repeatable conditions, which do not necessarily capture the practical use cases of the device. For instance, the British Hypertension Society and Association for the Advancement of Medical Instrumentation (AAMI) have specific protocols for testing cuff-based BP measurement devices^23^, which have been co-opted for cuffless BP estimation. However, the uncertainty estimated from the accuracy and precision obtained in specific tested situations, such as in-lab conditions, is unlikely to be reproducible in other, potentially more challenging, conditions. Since continuous BP monitoring is likely to be employed outside of the clinic, it is imperative to assign a confidence metric to each estimate based on the quality of the BP model inputs for outcome evaluation.

This paper focuses on (1) introducing a confidence metric that clinicians could use to discern data quality, and (2) using the confidence metric as an input to estimation fusion to improve BP estimation accuracy. To the authors’ knowledge, a confidence metric has never been used for cuffless BP estimation.

## Method

The method proposed herein is a three-step process. First, three BP estimation models are constructed and employed. Second, the uncertainty of the estimates for each model is evaluated. Finally, the three estimators and their uncertainties are fused, resulting in the final BP estimate and its associated uncertainty.

The prediction interval (PI) is used herein as a practical confidence metric since PI is concerned with the prediction accuracy of a target value^24^. PI is different from confidence interval (CI), which relates to the accuracy of our estimate of the true regression. Basically, PI considers more sources of uncertainty than CI, such as model error and noise variance^25^. In other words, CI is the uncertainty associated with the model parameters, whereas PI is the uncertainty associated with the estimation of future samples. The PI is, therefore, an approximation of the standard deviation of error in absence of the reference data.

A one-class support vector machine approach is investigated to estimate the PI of BP estimates. PI is then used as the input to our track fusion algorithm, which is the Covariance Intersection^26^. Covariance Intersection is employed to improve estimation accuracy by incorporating the estimators uncertainty in the fusion process. Different permutations of model fusion were studied to demonstrate the benefit of fusion on both BP and PI estimations. PI was finally used to flag BP estimates with large uncertainty to minimize the risk of false diagnosis due to erroneous estimates.

### Measurements

Five healthy participants (four males, age 28 ± 6.6 years) were recruited for data collection. All participants signed informed consent forms before participating in the study. Study protocols and procedures were all approved by the University of Waterloo (ORE#41490), Clinical Research Ethics Committee and conformed with the Declaration of Helsinki. This dataset is detailed in Landry et al.^7^ and is accessible through the IEEE DataPort (https://doi.org/10.21227/wysp-gt69). Briefly, continuous arterial BP waveform was measured using finger PPG (Portapres; Finapres Medical Systems, the Netherlands) and ECG and PPG were measured via the Astroskin wearable body metrics vest (Carré Technologies Inc., Canada). An iPhone SE was used to measure thigh orientation. All data were stored for processing at 64 Hz.

Testing sessions consisted of 6.5 hours of continuous BP monitoring. A 15-min procedure, used for model training, was performed at the beginning and again during the final 15 min of the data collection. The training procedure, designed to change the participant's BP within a range typical of activities of daily living, comprised a sequence of tasks included sitting, standing, walking, Valsalva maneuvers, and static handgrip exercises (for further details see Landry et al.^7^). During the six hours of testing, the participants were free to do as they pleased, excluding lying down and vigorous exercise.

### BP estimation models

#### NARX

A NARX architecture based on a multilayer Perceptron artificial neural network (ANN) was developed. The NARX model used exogenous inputs and BP at time-steps *k*-1 and *k*-2 to estimate BP at time point *k*. Exogenous inputs consisted of 0 to 18 time-step delays (19 samples) of the signals sampled at 64 Hz. The NARX input signals were ECG and a z-normalized PPG signal, the details of which, along with network training descriptions, can be found in Landy et al.^7^. The trained NARX model was found to accurately predict the BP waveform^7^, from which SBP and DBP were extracted for further analysis. To reduce the estimation variance, five NARX models were bootstrap-ensembled^27^, herein referred to as NARX.

#### ANN

Using the same ANN architecture, models were developed to estimate directly the SBP (ANN_Sys_) and the DBP (ANN_Dias_) from ECG and the z-normalized PPG signal (no BP estimates in the input layer). A similar model architecture is employed by Kachuee et al.^28^. Again, five ANN models were bootstrap-ensembled^27^, herein referred to as ANN_Sys_(ANN_Dias_).

#### PAT

According to Landry et al.^7^, the common logarithmic PAT model (PAT_Log_)^29^ is more accurate for estimating SBP during activities of daily living than the linear PAT model or the inverse quadratic PAT with linear HR term and is thus employed herein (referred to henceforth as PAT_Log_ and PAT refers to the actual time delay). PAT was computed using the Astroskin’s embedded algorithm, for which it is defined as the time difference between the ECG peak and 50% of the PPG amplitude (50% of foot-to-peak)^7^. PAT was post-processed at 256 Hz to improve temporal resolution compared to extracting PAT from the 64 Hz output data.

### Prediction interval estimation

In this section, support vector machines (SVMs) are used to estimate the PI. The general idea of SVM is to find the hyperplane that splits data into two classes with optimal margin, that is, the hyperplane with the largest distance to the closest data points (called support vectors)^30^. SVM utilizes a kernel to map input data into a high-dimensional feature space wherein the hyperplane is identified. When dealing with unlabeled data, one class SVM (OCSVM) can be used to find the underlying distribution of the data. In this scenario, the data can be separated into two categories according to a free parameter ν (0 < ν ≤ 1), instead of separating classes^31^. Note that a small value of ν leads to fewer support vectors and, therefore, a crude decision boundary^31^. In principle, OCSVM is a machine learning tool that splits the training data into “normal” and “outlier” categories using a hyperplane. The split is done according to a user-defined percentage of data intended to be outside the hyperplane. Thus, OCSVM is generally referred to as an outlier or a novelty detection algorithm^32^.

**Figure 1:**
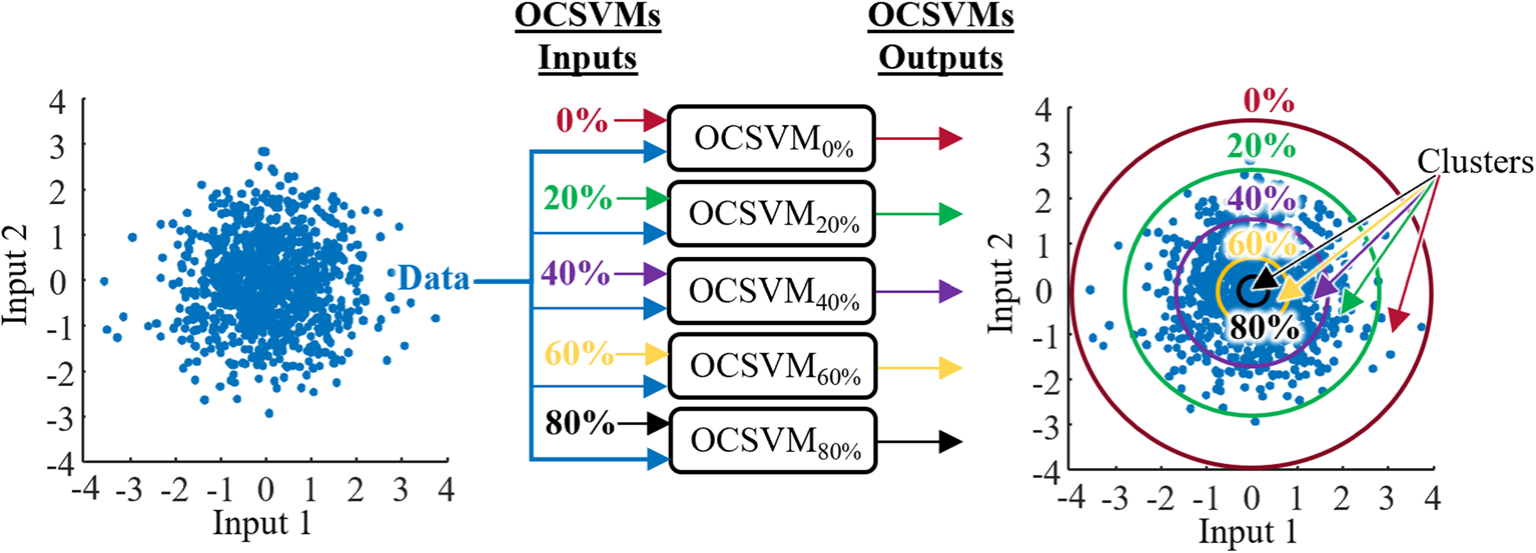
Prediction interval algorithm schematic featuring arbitrary data. Raw data are fed to multiple one-class support vector machine (OCSVM) models with their respective, user-defined, percentage of outliers. The outputs of the OCSVMs are hyperplanes that are projected on the two-dimensional input space (circles in this example). The clusters are upper and lower bounded by the hyperplanes (except inside 80% and outside 0%).

In this paper, a PI estimation model was trained independently for each individual’s SBP and DBP. The model architecture consisted of multiple OCSVMs trained on the two 15-min training sets using Gaussian kernels and a regularization parameter ν = 0.5. Each OCSVM was trained with all the training data, but with a different percentage of outliers from 0 to 99% in 1% increments. The 100 OCSVM hyperplanes were used to group data into 101 clusters, each spanning 10 hyperplanes (*i.e.*, between the 0% and the 9% hyperplanes, between the 1% and the 10% hyperplanes, etc.; note that from 91 to 99%, the clusters were defined as inside the X% hyperplane, instead of from X% to X + 9%). The data outside the 0% hyperplane was defined as the outlier cluster. A visual representation of the clustering method is shown in Fig. 1 with increments of 20% outliers for an arbitrary 2-dimensional input, illustrating initial raw data fed into multiples OCSVMS to compute hyperplanes, which are used for computing the clusters. For each cluster of training data, the BP estimation (SBP or DBP) error distribution was calculated, and the error standard deviation (${\upsigma }_{\mathrm{Err}}$) was associated with the uncertainty of that cluster. Therefore, any new datapoint assigned to a cluster was associated with that cluster’s uncertainty, herein referred to as the PI, in the testing phase. Clustering by intervals of 10 hyperplanes was used to increase the resolution of the estimated PI and ensure that ${\upsigma }_{\mathrm{Err}}$ was calculated on a sufficient number of datapoints.

Ten different inputs to the OCSVM were tested by varying the length of the input time series (ECG and PPG) in increments of 0.3 s (segment lengths were τ = 0.3*n* seconds long, where *n* $\in$ {1,2,3,4,5}) with (w/ BP) and without (w/o BP) the estimated BP waveform of length τ. The BP waveform was provided only for NARX. For ANN_Sys_(ANN_Dias_) and PAT_Log_, the estimated SBP (DBP) was provided instead.

### Estimation fusion

The covariance intersection method was used to fuse the BP estimates from the different models. For consistent estimates, A_i_ = {${\xi }_{{a}_{i}}$, ${P}_{{a}_{i}}$}, where $i$= 1 to $n$ number of estimates, and $\xi$ and $P$ are the mean and covariance of the estimate, respectively, the mean and covariance of the fused estimate C can be determined as

1 $${P}_{c}^{-1}={\omega }_{1}{P}_{{a}_{1}}^{-1}+\dots +{\omega }_{n}{P}_{{a}_{n}}^{-1}$$

2 $$\widehat{{\xi }_{c}}={P}_{c}\left({\omega }_{1}{P}_{{a}_{1}}^{-1}\widehat{{\xi }_{{a}_{1}}}+\dots +{\omega }_{n}{P}_{{a}_{n}}^{-1}\widehat{{\xi }_{{a}_{n}}}\right)$$

where ${\omega }_{i}$ are weights determined through an optimization process to minimize *P*_*c*_ and ${\sum }_{i=1}^{n}{\omega }_{i}=1$.

In this paper, $\xi$ refers to the estimated BP and $P$ is the PI estimated by the OCSVM model. Covariance intersection was used to fuse NARX and ANN_Sys_(ANN_Dias_) (NARX + ANN_Sys_(ANN_Dias_)), PAT_Log_ with other models (NARX + PAT_Log_ and ANN_Sys_(ANN_Dias_) + PAT_Log_), and for fusion of all three models (NARX + ANN_Sys_(ANN_Dias_) + PAT_Log_).

### Validation and data analysis

The 6 h of data between the two training procedures were used for testing the PI estimates of the models. For the NARX model, the estimated BP waveform was supplied to the OCSVM for training. For ANN_Sys_(ANN_Dias_) and PAT_Log_ models, only the estimated SBP(DBP) was fed to the OCSVM.

For each of the 10 PI model configurations, the MeRCI score^33^, defined as

3 $$\mathrm{MeRCI}^{\alpha} =\frac{1}{N}{\sum }_{i=1}^{N}{\lambda }^{\alpha }{\sigma }_{i}$$

was computed for the test dataset for each participant, where *N* is the number of SBP(DBP) datapoints, $\sigma$ is the BP estimated PI from the OCSVM model, and ${\lambda }^{\alpha }$ is obtained by first evaluating all ratios ${\lambda }_{i}=\left|{\widehat{y}}_{i}-{y}_{i}\right|/{\sigma }_{i}$ and then extracting the *α*th percentile of the ${\lambda }_{i}$ distribution; in this study the 99.7th percentile was used. The MeRCI score represents a mean scaled PI that contains *α*% of the data; thus, a smaller MeRCI score represents a better PI estimation. The MeRCI scores were calculated only for the BP estimates associated with a PI; i.e., if the BP estimate was classified outside the 0% hyperplane, the estimate was discarded.

The PI model configurations with the lowest MeRCI scores for SBP(DBP) were further investigated for each of the BP models. The Pearson correlation coefficients (*r*) were calculated for each participant to examine the linear relationship between ${\upsigma }_{\mathrm{Err}}$ of each cluster in the training and of the test data. The expected ${\upsigma }_{\mathrm{Err}}$ ± standard deviation (SD) in each cluster was also plotted against the percentage of outliers in the cluster.

The performance of the three BP models and the four fused models were analyzed using the mean error (${\upmu }_{\mathrm{Err}}$), ${\upsigma }_{\mathrm{Err}}$, mean absolute error (MAE), Pearson correlation coefficient, MeRCI score, and mean PI computed for the six-hour test data for each participant. To visualize each models performance in estimating changes in BP (ΔP), the mean absolute value of ΔP_model_ − ΔP_portapres_ was used (MAE_ΔP_), where ΔP is defined as the BP deviation from the mean and the subscript indicates whether the data are from the model or the Portapres device (herein considered the “ground truth”). The errors were separated into bins representing the distribution of the BP in the test dataset over the day. Each bin spanned a range of 5 mmHg, and the bins indicate deviations from the mean BP of the participant by 5* m* to 5(*m* + 1) mmHg, where *m* ranged from −6 to 6 for SBP and −4 to 4 for DBP. The difference of each bin with analogous bins from PAT_Log_ was computed as a function of |ΔP|. The previous step was done only for visualization purposes since PAT_Log_’s MAE_ΔP_ is not on the same scale as the other models, which made visualization difficult if not used as a reference. The MAEs were compared for each bin using a 2-way ANOVA test followed by a t-test with Bonferroni corrections to identify statistical differences across the models.

A standard deviation threshold (${\upsigma }_{\mathrm{T}}$) was used to filter the data, such that all BP estimates associated with a PI higher than ${\upsigma }_{\mathrm{T}}$ were removed from the dataset. For the remaining BP estimates (PI < ${\upsigma }_{\mathrm{T}}$), the percentage of data removed as a function of the absolute estimation error was computed for different ${\upsigma }_{\mathrm{T}}$. The ${\upsigma }_{\mathrm{Err}}$ and the percentage of data kept were computed for the six-hour test data to observe the tradeoff between the two. To visualize the error in estimating ΔP for the remaining data (PI < ${\upsigma }_{\mathrm{T}}$) in comparison with the initial dataset, MAE_ΔP_ as a function of |ΔP| was computed for ${\upsigma }_{\mathrm{T}}$ = 8 and 9 mmHg. To understand better the PI estimation performance, the percentage of data removed in each |ΔP| bin was computed. To assess the performance of the fused models during activities of daily living, ${\upsigma }_{\mathrm{Err}}$ was computed for different activities (sitting, standing, and walking), and compared with the value when using ${\upsigma }_{\mathrm{T}}$ = 8 mmHg using t-tests after validating data normality with one-sample Kolmogorov–Smirnov tests.

## Results

Herein only the results for SBP are presented for brevity. DBP results are included as supplementary material.

The MeRCI scores (mean ± SD) across the five subjects exhibited differences between the models, ranging from 24.4 for τ = 0.9 w/ BP (best) to 30.3 for τ = 0.3 w/o BP (worst) for NARX, from 24.9 for τ = 0.9 w/ BP (best) to 28.4 for τ = 1.5 w/o BP (worst) for ANN_Sys_, and from 28.0 for τ = 0.6 w/ BP (best) to 33.1 for τ = 0.3 w/o BP (worst) for PAT_Log_.

**Figure 2:**
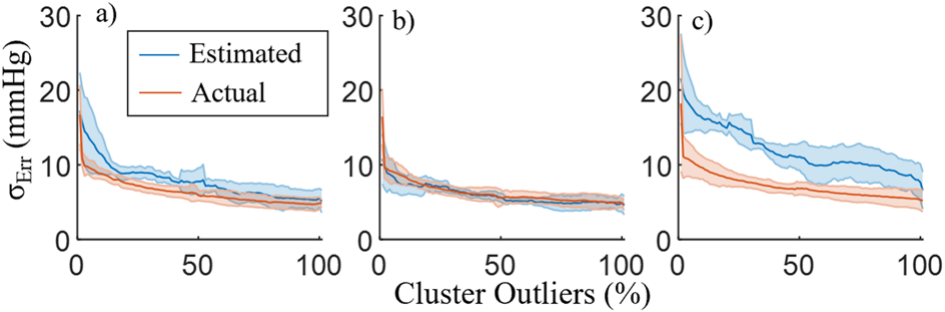
The SBP estimated and actual data standard deviation of error in every cluster for (**a**) NARX, (**b**) ANN_Sys_, and (**c**) PAT_Log_. Lines represent the mean of all subjects, and the error bars show ± SD.

The MeRCI score for PAT_Log_ using the τ = 0.9 w/ BP model was 28.5, only slightly higher than its best configuration (τ = 0.6 w/ BP); therefore, we use the PI model τ = 0.9 w/ BP for the three BP models for simplicity of comparison for the remainder of the analysis. The estimated and actual clusters’ ${\upsigma }_{\mathrm{Err}}$ as a function of the clusters’ percentage of outliers (lower bound) are shown in Fig. 2 for the NARX, ANN_Sys_, and PAT_Log_ models. Note that the ${\upsigma }_{\mathrm{Err}}$ on the training data (in blue) are used to estimate the testing data ${\upsigma }_{\mathrm{Err}}$ (in red). Therefore, Fig. 2 compares the estimated and the actual ${\upsigma }_{\mathrm{Err}}$ during activities of daily living. The over/underestimation of ${\upsigma }_{\mathrm{Err}}$ in each cluster reflects the three BP models’ difference in precision on the training vs test data. The difference in the ${\upsigma }_{\mathrm{Err}}$ evaluated on all the training data compared to the ${\upsigma }_{\mathrm{Err}}$ evaluated on all the test data was, on average, 17%, -22% and 82%, for NARX, ANN_Sys_, and PAT_Log_, respectively. Note that the training ${\upsigma }_{\mathrm{Err}}$ at 0% of outliers is not defined, as there are no training data in this cluster. In the test data, the data points outside the 0% hyperplane are outliers and represent 3.9 ± 2.0%, 3.3 ± 1.3% ,and 4.9 ± 4.2% of the BP estimates for the NARX, ANN_Sys_, and PAT_Log_ models, respectively. The Pearson correlation coefficients between the training and testing ${\upsigma }_{\mathrm{Err}}$ of each cluster are *r* = 0.88 ± 0.11, *r* = 0.81 ± 0.09, and *r* = 0.86 ± 0.13 for the NARX, ANN_Sys_, and PAT_Log_ models, respectively. It can be observed in Fig. 2 that the model overestimates, on average, ${\upsigma }_{\mathrm{Err}}$ of the test data by 21% and 68% for NARX and PAT_Log_, respectively, and underestimates it by 3% for ANN_Sys_.

**Figure 3:**
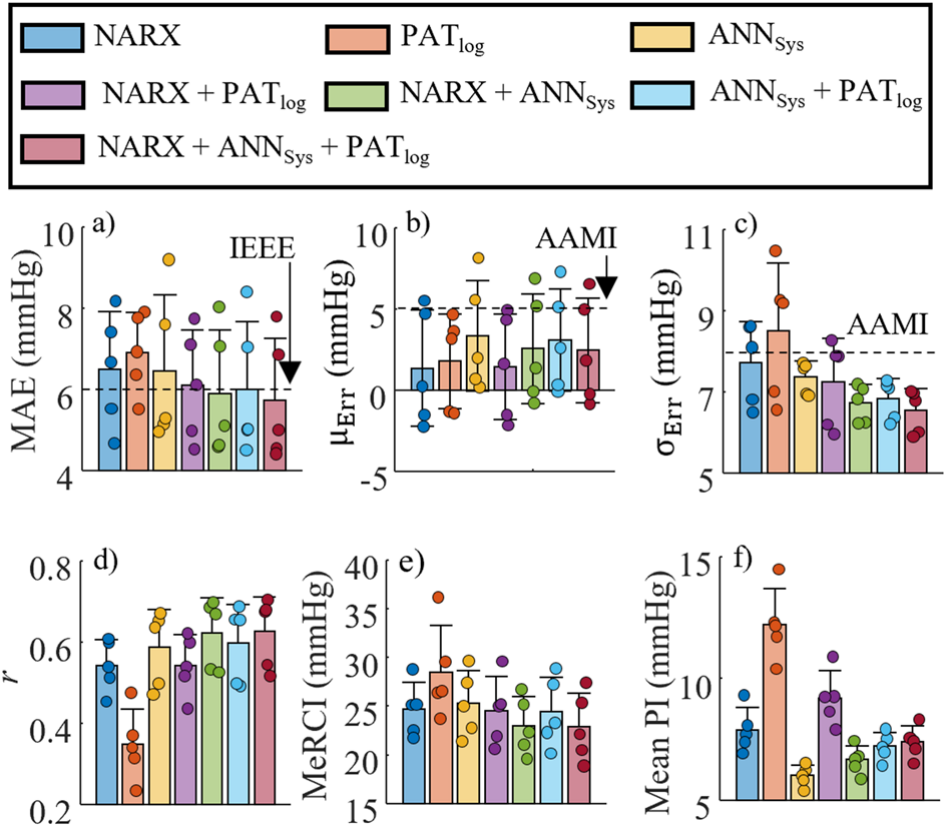
Comparison of (**a**) mean absolute error, (**b**) mean error, (**c**) standard deviation of the error, (**d**) Pearson correlation coefficient between different model estimates and the BP measurements, (**e**) MeRCI score, and (**f**) mean estimated prediction interval. Bars represent the mean of all subjects and the error bars show ± SD. Each data point represents one participant. The IEEE cuffless wearable and the AAMI standard limits are also shown via horizontal dashed lines in subplots (**a**), and (**b,c**), respectively.

The mean and SD of the MAE, ${\upmu }_{\mathrm{Err}}$, ${\upsigma }_{\mathrm{Err}}$, *r*, MeRCI score, and mean of the PI during activities of daily living computed across the five subjects are shown in Fig. 3. The results exhibit differences in mean MAE between the models, ranging from 5.74 mmHg for NARX + ANN_Sys_ + PAT_Log_ (best) to 6.91 for PAT_Log_ (worst). The IEEE cuffless wearable standard limit^34^, which is met by all fused models except NARX + PAT_Log_ and by none of the initial models, is also shown. The horizontal dashed lines in Fig. 3b indicate the AAMI requirement limit for ${\upmu }_{\mathrm{Err}}$ ^35^, which is met by all models. All models meet the ${\upsigma }_{\mathrm{Err}}$ AAMI standard except for PAT_Log_. It is noted that ${\upsigma }_{\mathrm{Err}}$ is under 8 mmHg for all participants except for NARX, PAT_Log_, and NARX + PAT_Log_. The average Pearson correlation coefficient between the estimated and measured SBP for all five participants obtained with the fusion of at least the two ANN models (NARX and ANN_Sys_) is higher than the other models. The MeRCI score shows that model fusion improves PI estimation when compared to the three initial non-fused models. ANN_Sys_ has the lowest mean PI, but a larger MeRCI score than the other fused models, indicating that it underestimates the PI.

**Figure 4:**
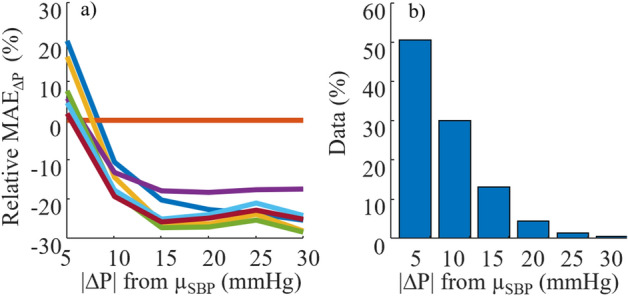
(**a**) Mean of SBP MAE_ΔP_ relative to MAE_ΔP_ – PAT_Log_ for all participants, and (**b**) the distribution of data (all participants aggregated) against the distribution of |∆P| from µ_SBP_ measured throughout the day. The MAE_ΔP_ are binned in increments of 5 mmHg from −30 to 30 mmHg then grouped according to their absolute value. Legend according to Fig. 3.

The difference between MAE_ΔP_ of each model and the MAE_ΔP_ of PAT_Log_ (in percentage) as a function of absolute BP deviation from the mean is shown in Fig. 4a; the data distributions when aggregating the data of all participants are included in Fig. 4b. The range of SBP shown is the minimum range spanned by all participants.

**Figure 5:**
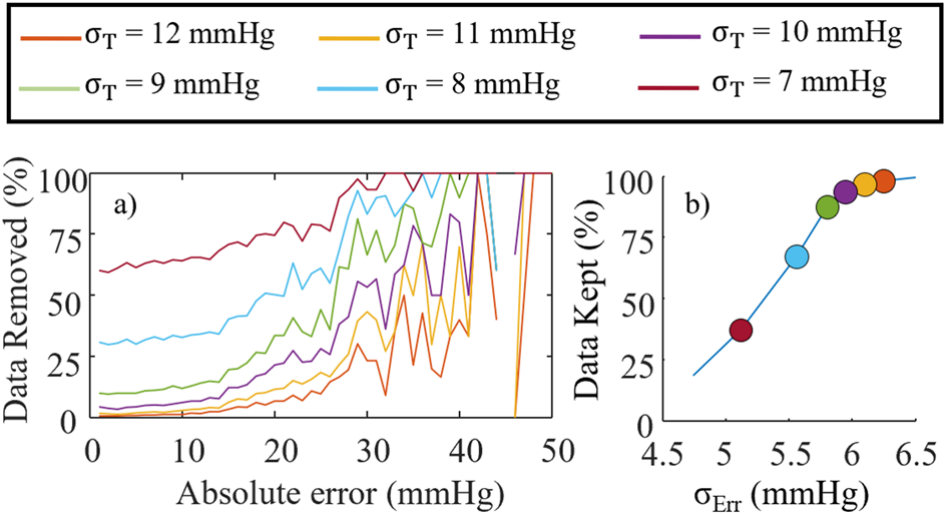
For the NARX + ANN_Sys_ + PAT_Log_ model, (**a**) percentage of the data removed as a function of the absolute error using different ${\upsigma }_{\mathrm{T}}$. The results are calculated from all the test data from every participant and grouped in increments of 1 mmHg. (**b**) Percentage of data kept as a function of the error standard deviation when varying the threshold on the prediction interval. The line was computed by varying the ${\upsigma }_{\mathrm{T}}$, where specific values are marked with circles according to the legend.

The percentage of data removed is plotted against the absolute estimation error in Fig. 5a for the NARX + ANN_Sys_ + PAT_Log_ model. The tradeoff between the amount of data retained and ${\upsigma }_{\mathrm{Err}}$ when varying ${\upsigma }_{\mathrm{T}}$ is shown in Fig. 5b.

**Figure 6:**
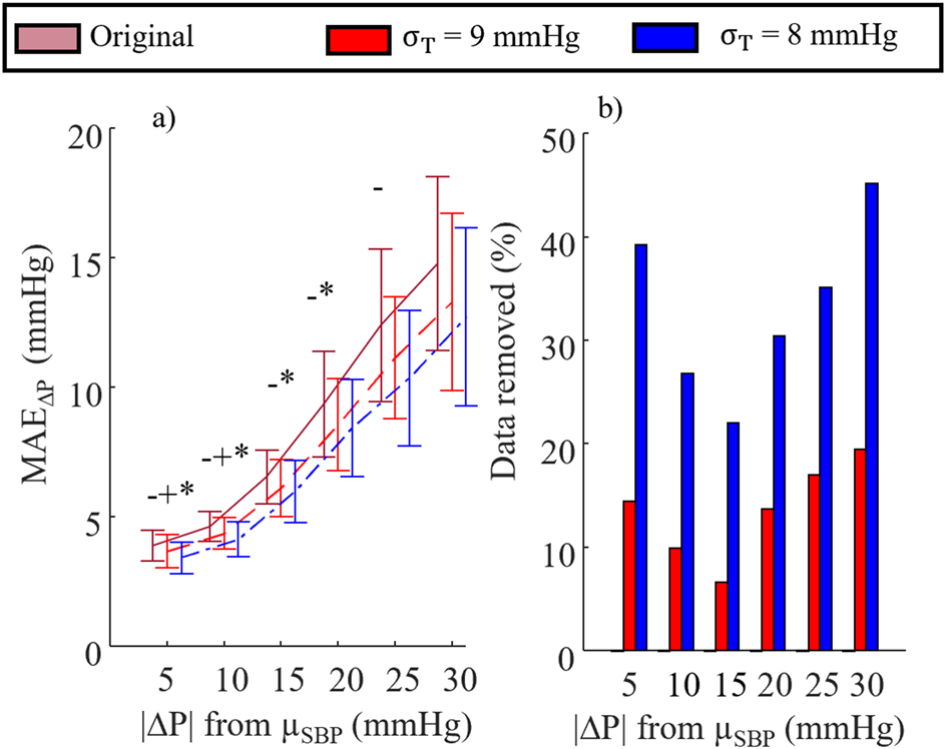
For the NARX + ANN_Sys_ + PAT_Log_ model, (**a**) mean and standard deviation of MAE_ΔP_ for all participants, and (**b**) percentage of data removed from each bin versus |∆P| from µ_SBP_ measured throughout the day using different threshold (${\upsigma }_{\mathrm{T}}$). In (**a**) lines represent the mean of all subjects and the error bars show ± SD. t-test with p < 0.05/3 (*). Original vs ${\upsigma }_{\mathrm{T}}$ = 9 mmHg, (−), original vs ${\upsigma }_{\mathrm{T}}$ = 8 mmHg, and ( +) ${\upsigma }_{\mathrm{T}}$ = 8 mmHg vs ${\upsigma }_{\mathrm{T}}$ = 9 mmHg (p < 0.05/3).

MAE_ΔP_ as a function of |ΔP| is plotted in Fig. 6a for the NARX + ANN_Sys_ + PAT_Log_ model using ${\upsigma }_{\mathrm{T}}$ = 8 and 9 mmHg. The percentages of data removed in each |ΔP| bin are plotted in Fig. 6b. When using the OCSVM model of PI to remove data with large expected error SD (PI ≥ 8 or 9 mmHg), all expected MAE_ΔP_ values are lower than their original values. In every case, the BP estimates by the fused model at large |ΔP| have higher expected error than BP estimates closer to the mean BP. There were, however, statistical differences when comparing each bin; randomly removing the same amount of data in the dataset did not result in the observed reduction in MAE_ΔP_.

**Figure 7:**
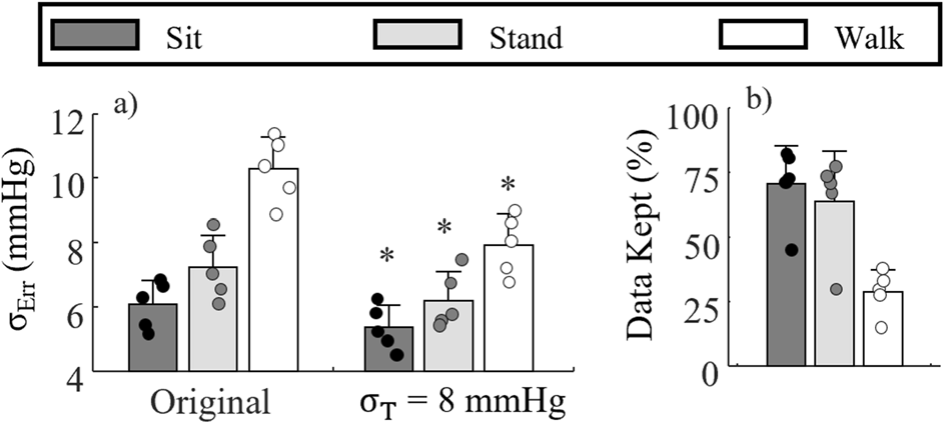
(**a**) Comparison of error standard deviation for the original NARX + ANN_Sys_ + PAT_Log_ model and the same model using a ${\upsigma }_{\mathrm{T}}$ = 8 mmHg during sitting, standing, and walking, and (**b**) percentage of data kept (prediction interval < 8 mmHg). Bars represent the mean of all subjects and the error bars show ± SD. Each data point represents one participant. (*) t-test with p < 0.05 with respect to their original counterpart.

The ${\upsigma }_{\mathrm{Err}}$ for the NARX + ANN_Sys_ + PAT_Log_ model is shown in Fig. 7a when estimating BP during sitting, standing, and walking. The ${\upsigma }_{\mathrm{Err}}$ is shown for original beat-by-beat estimation and for the ${\upsigma }_{\mathrm{T}}$ = 8 mmHg case; the percentage of data kept for each activity is shown in Fig. 7b. It can be seen that estimating BP during walking is difficult. The expected ${\upsigma }_{\mathrm{Err}}$ of NARX + ANN_Sys_ + PAT_Log_ is 10.3 mmHg, which is lower by 0.6 mmHg, 1.6 mmHg, and 3.9 mmHg, than the NARX, ANN_Sys_, and PAT_Log_, models, respectively. Moreover, the expected ${\upsigma }_{\mathrm{Err}}$ decreases to 7.95 mmHg when using a ${\upsigma }_{\mathrm{T}}$ = 8 mmHg threshold on the PI, and the expected mean error is down to 1.7 mmHg (t-test p < 0.05). The previous results show that using the fusion approach presented herein results in BP estimates during walking achieving the AAMI standard 29% of the time (Fig. 7b). Using ${\upsigma }_{\mathrm{T}}$ = 9 mmHg increases the error SD to 8.3 mmHg while 53% of data are kept. Estimating BP while sitting and standing is an easier task, as can be observed with their respectively lower ${\upsigma }_{\mathrm{Err}}$ values in comparison with walking. However, they still benefit by using a ${\upsigma }_{\mathrm{T}}$ = 8 mmHg threshold, which decreases their ${\upsigma }_{\mathrm{Err}}$ by 12% and 14%, respectively (both t-tests p < 0.05), while keeping 71% and 64% of the BP estimates, respectively.

## Discussion

This work was motivated by two needs: (1) to improve cuffless BP estimation, and (2) to provide a measure of the degree of confidence a practitioner should have in the BP estimates. The PI model architecture using OCSVM presented herein can be applied to different BP models, from simple PAT models to more complex NARX models. The Covariance intersection fusion algorithm is also generic since it does not rely on any assumptions on the variables to be fused; only their means and covariances are needed. The method developed herein is not limited in terms of the number of models that can be fused. However, the Covariance intersection algorithm will become more complex to optimize as the number of models increases^36^. Herein, we used a small dataset specifically designed to train subject-specific BP models; however, the method is also applicable to population-based models. The one modification that would be needed for Big Data is to add kernel approximation techniques to minimize the training time of the OCSVMs^37^. Further studies are required to assess the fusion of population-based models.

In this study, the data of two 15-min training procedures separated by a six-hour testing time window were used to train the models. The test period was limited to six hours for practical reasons, namely recruiting participants. In real-life scenarios, this period could be extended without affecting the models accuracy. Therefore, a clinician could obtain a whole day (nighttime excluded) of BP data by collecting only 15-min data segment at the beginning and at the end of the day, which is deemed reasonable, practical and useful. While a single calibration utilizing only a single data segment at the beginning of the day would have practical benefits, preliminary studies showed an increase in model performance by using two data segments performed at different times of the day^14^. The hypothesis therein was the model is better able to capture changes in the physiological state of the person and/or sensor position. Nevertheless, the intended goal of this monitoring system was for a clinician to post review the data, making sure the blood pressure measurements are accurate and reliable. Therefore, having the extra training session at the end of the day, although it hinders the access to real-time estimations of BP, is not detrimental to the goal of this study.

Herein, we have shown that the PI estimation varied depending on the inclusion of estimated BP as an input to the OCSVMs, and the length of the input signals (ECG, PPG, and BP). The obtained MeRCI scores, indicating how well the PI represented the observed error^33^, demonstrate the importance of using estimated BP as an input to the PI model. This pilot testing showed that 0.9 s of data (τ = 0.9) yielded the most accurate estimation of ${\upsigma }_{\mathrm{Err}}$ and, therefore, PI for the models for every cluster indicating sufficient information encoded in that duration. The lower performance when using more than 0.9 s is probably caused by overfitting of the OCSVMs to the training data due to too many parameters of the model.

High Pearson correlation coefficients for the NARX, ANN_Sys_, and PAT_Log_ models have been observed between the estimated and actual PI for each cluster. This shows the validity of using ${\upsigma }_{\mathrm{Err}}$ from the training data clusters as a surrogate for the PI when estimating BP, as is typically done in clustering-based PI computations^38^. The over/underestimation of ${\upsigma }_{\mathrm{Err}}$ in each cluster can be explained by the difficulty of estimating the training dataset and by how each model is trained. The 15-min procedures performed by the participants to train the models were designed to create large BP variability in a short amount of time. This makes it a short but difficult time series to estimate for PAT_Log_, which has only two tunable parameters, in comparison with the ANN_Sys_ (780 parameters) and NARX (820 parameters) models. NARX has more parameters, but it is trained in a feedforward fashion, and predicts as a recurrent ANN, which makes the prediction of the training data more difficult compared to ANN_Sys_. Apart from over/underestimating, all models exhibit negative trends in Fig. 2, which shows that the further away from the center of the training dataset is a BP estimate, the more likely the estimation error will be large. This highlights the importance of using an outlier detection algorithm, such as OCSVM, to cluster data.

The estimated ${\upsigma }_{\mathrm{Err}}$ of the BP was smaller when fusing all three models (Fig. 3c). On average, the NARX + ANN_Sys_ + PAT_Log_ model decreased ${\upsigma }_{\mathrm{Err}}$ by 1.2 mmHg, 0.8 mmHg, and 2.0 mmHg over the individual NARX, ANN_Sys_, and PAT_Log_ models, respectively, with a consistent decrease for every participant. The ${\upmu }_{\mathrm{Err}}$, however, is slightly higher when compared with NARX and PAT_Log_ due to the fusion with ANN_Sys_, which is more biased than the other estimators (Fig. 3b). Nevertheless, MAE is the lowest when fusing all the models (Fig. 3a). A simple approach to decrease the bias of the fused models would be to correct its mean estimation to that of the least biased model (PAT_Log_). However, a larger sample size would be required to validate that PAT_Log_ is the least biased.

Using PAT_Log_ in the fusion process is beneficial for the activities of daily living dataset because the model is good at estimating small change in BP (Fig. 4a); for this dataset, as observed in Fig. 4b, about 50% of the time BP fluctuates between ± 5 mmHg of the mean BP. The NARX + ANN_Sys_ + PAT_Log_ model benefits from this and performed similarly to PAT_Log_ in that range of ΔP. Fusion of the three models is beneficial up to a ΔP of ± 10 mmHg, where NARX + ANN_Sys_ + PAT_Log_ model outperformed the other models for 80% of the data (except PAT_Log_ for ΔP of ± 5 mmHg), explaining its best overall MAE. This result, however, will be highly dependant on the distribution of BP that is to be estimated. With a larger change in BP, NARX + ANN_Sys_ might be more suited for the task.

Estimation of PI is best when using both NARX + ANN_Sys_ and NARX + ANN_Sys_ + PAT_Log_ models based upon the MeRCI scores. Mean PI is, however, lower for NARX + ANN_Sys_. Estimation of the degree to which PI is over/underestimated can be computed by dividing the MeRCI score by the mean PI, which should give 3 for an ideal PI when computing the MeRCI score with 99.7% of the ${\lambda }_{i}$ distribution (99.7% of the data of a normal distribution should be covered by ± 3 SD). For the NARX + ANN_Sys_ model, this ratio is 3.5, thus, on average the PI is underestimated by 16%. For the NARX + ANN_Sys_ + PAT_Log_ model, the mean underestimation is 4%. This computation is a crude estimation of the goodness of the PI since it only considers the mean PI and MeRCI score, and not the PI estimation accuracy at different ranges of PI and ΔP. Nevertheless, it was shown that the estimated PI could be effectively used to remove large errors from the BP estimation time series. However, it comes with a compromise between removing BP estimates with low and high errors for different ${\upsigma }_{\mathrm{T}}$, as shown in Fig. 5a. This compromise is, however, successful in decreasing ${\upsigma }_{\mathrm{Err}}$ as ${\upsigma }_{\mathrm{T}}$ is decreased (Fig. 5b). Moreover, the PI model is successful in decreasing ${\upsigma }_{\mathrm{Err}}$ for every individual.

The goal of this study is to provide an uncertainty measure for the BP estimates and to remove the problematic data that can confuse a diagnosis (to make the cuffless measurement more reliable). As shown in Fig. 6a, an increase in the BP variation intrinsically increases the error the model in the estimating BP; therefore, one might expect that OCSVMs cluster the data by range of BP and, when using a ${\upsigma }_{\mathrm{T}}$ = 9 (or 8) mmHg threshold on the PI, it would remove only estimates at both ends of the BP range. However, this is not the case, as observed in Fig. 6a, the PI estimation model decreased the error for the whole range of BP, and removed data from every |ΔP| bin (Fig. 6b). To evaluate the success of the proposed method for removing outliers, one should: i) compare the estimates after the removal of samples with the original estimates (Fig. 6a, pink vs blue and red); ii) look at the percentage of removed data points with the proposed method with the threshold of 9 (or 8) mmHg, which is hardly beyond 15% (35%) of data and represent a nonmonotonic behavior with respect to the variance of BP indicating that the variation in BP is not the only factor in removing more samples, but the samples are removed objectively to lower the error of the fused models.

Figure 7 shows the PI model capability to remove BP estimates with large errors for different activities of daily living. For the OCSVMs, there is no distinction between motion artefacts and estimation error due to model inaccuracy since the PI model only learns uncertainty based on the statistical error distribution from the training data. In other words, large uncertainty, whether from noise or model inaccuracy, leads to larger estimation errors, and those data points should be discarded before diagnosis. For instance, estimation of BP during walking was not precise due two main factors. First, the BP waveforms are distorted due to, both, the interaction between the heart’s pressure wave and the wave generated by the whole body vertical movement^39^. The latter is difficult for the BP model to capture. Second, the presence of motion artefacts in the PPG and ECG signals creates artefact in the BP estimation. While it was not the focus of this research, the motion artefacts can be filtered out using movement information acquired from wearable inertial measurement units^40^. In such cases, the PI model will learn the error distribution based on the filtered signal, and therefore the underlying error distribution with the removed effect of artefacts.

A limitation of this study is the that the number of subjects is somewhat small. We note, however, that the validation for each subject extends for 6 h, with only 15 min of training at the beginning and 15 min of training at the end of the session. The method detailed herein to estimate BP shows great potential for improving continuous BP estimation by filtering out BP estimates with large uncertainty. Despite the relatively small sample size, we showed in Fig. 7a statistical improvement in the BP estimation by using this method”.

An ideal PI model would, when selecting ${\upsigma }_{\mathrm{T}}$, result in no individual ${\upsigma }_{\mathrm{Err}}$ larger than ${\upsigma }_{\mathrm{T}}$ in every activity/posture. This was not the case with the NARX + ANN_Sys_ + PAT_Log_ model as it could not reach 8 mmHg for every participant while walking (Fig. 7), although it reaches that level of error on average and for every participant while sitting and standing. This shows that the approach presented herein is a viable solution for cuffless BP estimation to be used in clinical settings as the BP estimates with a PI lower than 8 mmHg will meet the AAMI standard independent from the conditions in which the BP is measured. In the worst-case scenario, there are no high-quality BP estimates (PI < 8 mmHg), but at least erroneous estimates would not be mistakenly used for diagnosis.

## Conclusion

This paper examined the estimation fusion of up to three state-of-the-art models to achieve better cuffless BP estimation accuracy. We emphasized the importance of the prediction interval in the process of fusion, also in the context of clinical use of the estimated BP data, for which high quality estimates are essential. For that purpose, a clustering approach using one-class support vector machines was developed to estimate the prediction interval independent of the model architecture.

It was shown that the prediction interval model can cluster the BP estimates in terms of their quality, which was quantified by the error standard deviation and used to estimate the prediction interval. The prediction interval model was used with the three different BP estimation models (NARX, ANNSys, and PATLog), and the results were fused using the covariance intersection fusion algorithm. The fusion of the three models showed improvement in BP estimation accuracy as well as in its estimated prediction interval.

Although this work went throught a very technical method to demonstrate the importance of PI, the essence of the work is simple; PI (or other confidence metrics) should be used to assess the quality of cuffless BP estimate. Withtout it, there is no way to ensure that the BP estimates represent well the actual BP of an individual during daily life, risking erroneous clinical evaluation.

## Supplementary Information

Supplementary Figures.

## Data Availability

Data are available on IEEE DataPort under “Wearable Physiological and Blood Pressure Measurements During Activities of Daily Living”.
