# Supplementary material for: A fusion approach to improve accuracy and estimate uncertainty in cuffless blood pressure monitoring
Source: Sci Rep. 2022 May 13;12:7948. doi: 10.1038/s41598-022-12087-7 (PMC9106676; doi:10.1038/s41598-022-12087-7)
Supplement: Supplementary file 1 — Supplementary Figures. [file 41598_2022_12087_MOESM1_ESM.docx]

A Fusion Approach to Improve Accuracy and Estimate Uncertainty in Cuffless Blood Pressure Monitoring – Supplementary Material

Cederick Landry, Sean D. Peterson, and Arash Arami

The main manuscript presents results for systolic blood pressure. Herein we supplement those results with analogous findings for diastolic blood pressure (DBP).

The training and testing clusters’ error standard deviation ($\sigma_{\mathrm{Err}}$) for the NARX, ANN_Dias_, and PAT_Log_ models are shown in Figure S1 as a function of the clusters’ percentage of outliers (lower bound) . Note that the training $\sigma_{\mathrm{Err}}$ at 0% of outliers is not defined, as there are no training data in this cluster. In the test data, those are outliers and represent 3.1±1.3%, 4.3±1.7%, and 4.8±3.4% of the BP estimates for the NARX, ANN_Dias_, and PAT_Log_ models, respectively. The Pearson correlation coefficients between the training and testing $\sigma_{\mathrm{Err}}$ of each cluster are *r* = 0.80±0.24, 0.72±0.23, and 0.78±0.11 for the NARX, ANN_Dias_, and PAT_Log_ models, respectively. It can be observed in Figure S1 that the model overestimates on average the $\sigma_{\mathrm{Err}}$ of the test data by 43%, 13%, and 57% for NARX, ANN_Dias_, and PAT_Log_, respectively.


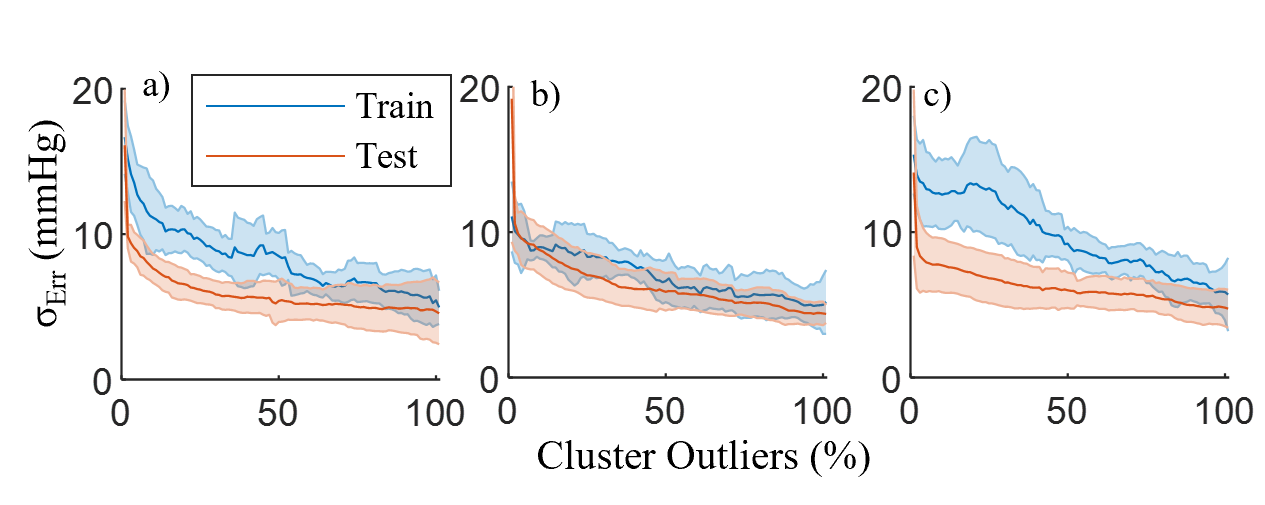


Figure S1. The diastolic blood pressure training and testing data standard deviation of error in every cluster for a) NARX, b) ANN_Dias_, and c) PAT_Log_. Lines represent the mean of all subjects and the shaded region show ± SD.

The mean and standard deviation (SD) of the MAE, mean error ($\mu_{\mathrm{Err}}$), $\sigma_{\mathrm{Err}}$, *r*, MeRCI score, and mean of the prediction interval (PI) during activities of daily living computed across the five subjects are shown in Figure S2.


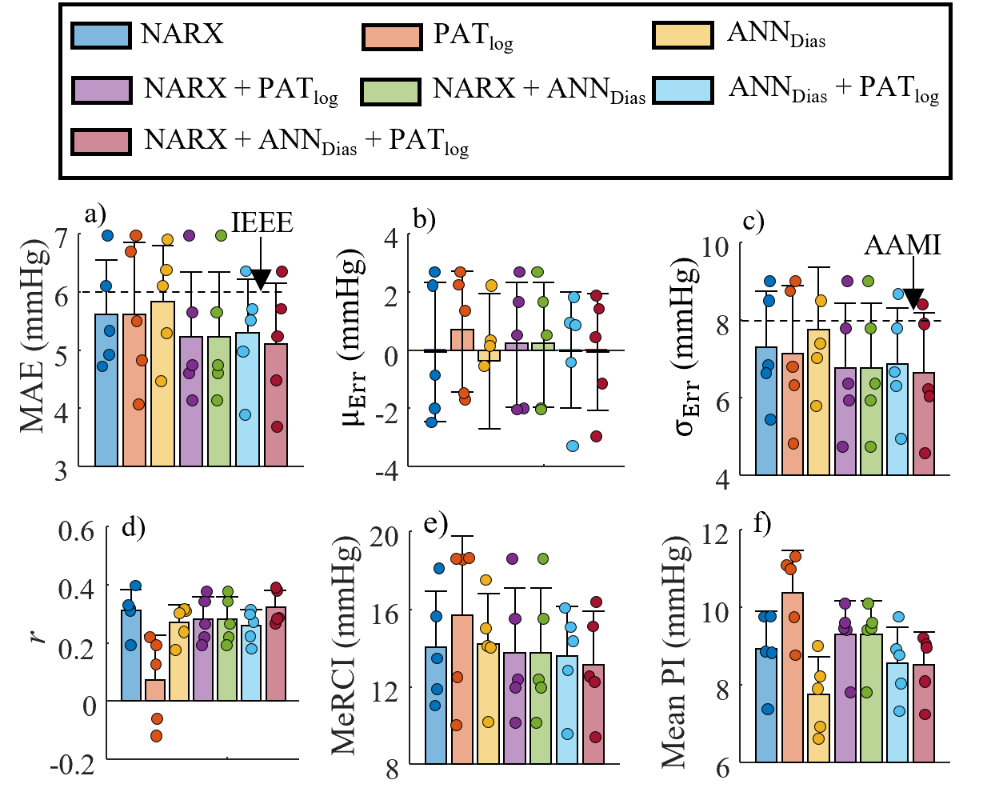


Figure S2. Comparison of a) mean absolute error, b) mean error, c) standard deviation of the error, d) Pearson correlation coefficient between different model estimates and the BP measurements, e) MeRCI score, and f) the mean estimated prediction interval. Bars represent the mean of all subjects and the error bars show ± SD. Each data point represents one participant. The IEEE cuffless wearable and the AAMI standard limits are also shown in subplots a), and b-c), respectively.


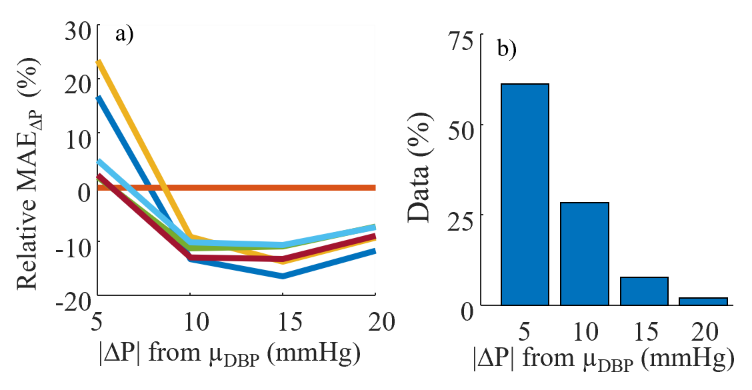


Figure S3 a) Mean of the DBP MAE_ΔP_ relative to MAE_ΔP_ – PAT_Log_ for all participants, and b) the distribution of data of all participants aggregated against the distribution of |∆P| from µ_SBP_ measured throughout the day. The MAE_ΔP_ are binned in increments of 5 mmHg from -20 mmHg to 20 mmHg then grouped together according to their absolute value.

Figure S3.a depicts the difference in MAE_ΔP_ of each bin with analogous bins from PAT_Log_ was computed as a function of |ΔP|; the data distributions when aggregating the data of all participants are included in Figure S3.b. The range of DBP shown is the minimum range spanned by all the participants.

The percentage of data removed is plotted against the absolute estimation error in Figure S4.a for the NARX + ANN_Dias_ + PAT_Log_ model. The tradeoff between amount of data kept and $\sigma_{\mathrm{Err}}$ when varying threshold values $\sigma_{T}$ for NARX + ANN_Dias_ + PAT_Log_ is shown in Figure S4.b.


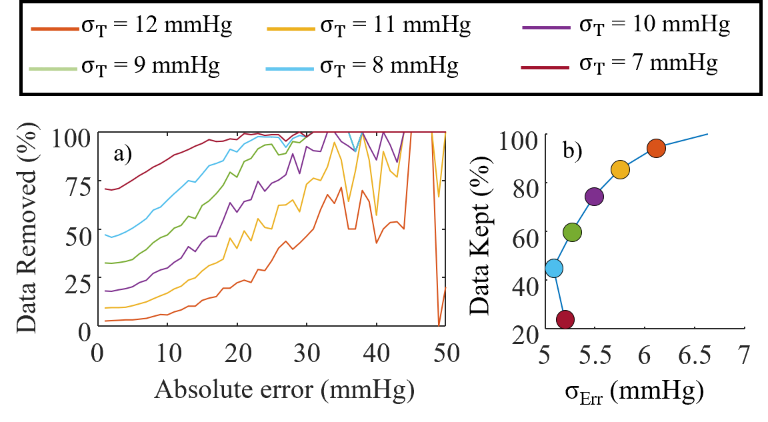


Figure S4. a) Percentage of the data removed as a function of the absolute error using different thresholds $\sigma_{T}$. The results are calculated from all the test data from every participant and grouped in increments of 1 mmHg. b) Percentage of data kept as a function of the error SD when varying the threshold on the PI. The line was computed by varying the $\sigma_{T}$, where specific values are marked with circles according to the legend.


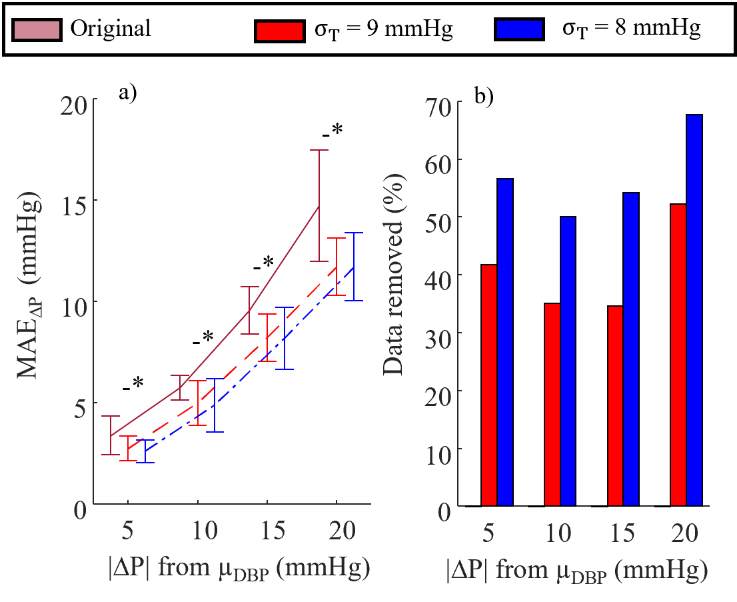


Figure S5. a) Mean and SD of MAE_ΔP_ for all participants, and b) percentage of data removed from each bin, against the |∆P| from µ_DBP_ measured throughout the day. In a) bars represent the mean of all subjects and the error bars show ± SD. Legend: (*) Original vs $\sigma_{T}$ = 9 mmHg, (-) Original vs $\sigma_{T}$ = 8 mmHg, and (+) $\sigma_{T}$ = 8 mmHg vs $\sigma_{T}$ = 9 mmHg (p<0.05/3)

The MAE_ΔP_ as a function of |ΔP| is plotted in Figure S5.a for the NARX + ANN_Dias_ + PAT_Log_ model using $\sigma_{T}$ = 8 and 9 mmHg. The percentage of data removed in each |ΔP| bin is plotted in Figure S5.b. All the expected MAE_ΔP_ values are lower when using the OCSVM model PI to remove data with large expected error SD (PI ≥8 or 9 mmHg). In every case, the BP estimations at large |ΔP| by the fused model have a higher expected error than BP estimations closer to the mean BP.

The error SD for the NARX + ANN_Dias_ + PAT_Log_ model is shown in Fig S6.a when estimating BP during sitting, standing, and walking. The $\sigma_{Err}$ is shown for original beat-by-beat estimation and for the SD threshold $\sigma_{T}$ = 8 mmHg case. The percentage of data kept for each activity when using $\sigma_{T}$ = 8 mmHg is shown in Figure S6.b.


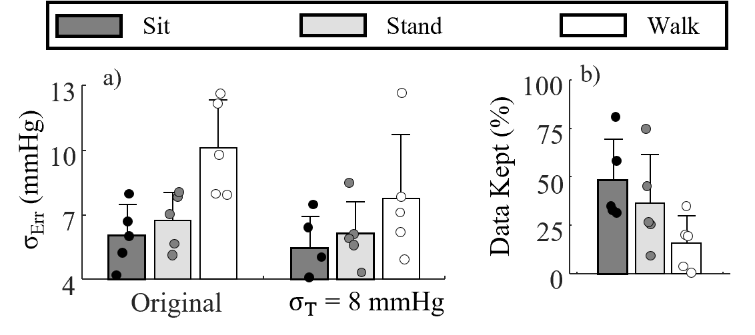


Figure S6. a) Comparison of the $\sigma_{Err}$ for the original NARX + ANN_Dias_ + PAT_Log_ model and the same model using $\sigma_{T}$ = 8 mmHg during sitting, standing, and walking, and b) the percentage of data kept (PI < 8 mmHg). Bars represent the mean of all subjects and the error bars show ± SD. Each data point represents one participant.
